# Pollinator and habitat-mediated selection as potential contributors to ecological speciation in two closely related species

**DOI:** 10.1093/evlett/qrad060

**Published:** 2023-11-23

**Authors:** Diane R Campbell, John M Powers, Madison Crowell

**Affiliations:** Department of Ecology and Evolutionary Biology, University of California, Irvine, CA, United States; Rocky Mountain Biological Laboratory, Crested Butte, CO, United States; Department of Ecology and Evolutionary Biology, University of California, Irvine, CA, United States; Rocky Mountain Biological Laboratory, Crested Butte, CO, United States; Rocky Mountain Biological Laboratory, Crested Butte, CO, United States

**Keywords:** cline, divergent selection, floral traits, pollination, speciation, vegetative traits

## Abstract

In ecological speciation, incipient species diverge due to natural selection that is ecologically based. In flowering plants, different pollinators could mediate that selection (pollinator-mediated divergent selection) or other features of the environment that differ between habitats of 2 species could do so (environment-mediated divergent selection). Although these mechanisms are well understood, they have received little rigorous testing, as few studies of divergent selection across sites of closely related species include both floral traits that influence pollination and vegetative traits that influence survival. This study employed common gardens in sites of the 2 parental species and a hybrid site, each containing advanced generation hybrids along with the parental species, to test these forms of ecological speciation in plants of the genus *Ipomopsis*. A total of 3 vegetative traits (specific leaf area, leaf trichomes, and photosynthetic water-use efficiency) and 5 floral traits (corolla length and width, anther insertion, petal color, and nectar production) were analyzed for impacts on fitness components (survival to flowering and seeds per flower, respectively). These traits exhibited strong clines across the elevational gradient in the hybrid zone, with narrower clines in theory reflecting stronger selection or higher genetic variance. Plants with long corollas and inserted anthers had higher seeds per flower at the *Ipomopsis tenuituba* site, whereas selection favored the reverse condition at the *Ipomopsis aggregata* site, a signature of divergent selection. In contrast, no divergent selection due to variation in survival was detected on any vegetative trait. Selection within the hybrid zone most closely resembled selection within the *I. aggregata* site. Across traits, the strength of divergent selection was not significantly correlated with width of the cline, which was better predicted by evolvability (standardized genetic variance). These results support the role of pollinator-mediated divergent selection in ecological speciation and illustrate the importance of genetic variance in determining divergence across hybrid zones.

## Introduction

The formation of new species involves divergence of phenotypes and development of reproductive isolation (Dobzhansky, 1937). In ecological speciation, the initial divergence of phenotypes results from divergent natural selection, and reproductive isolation arises directly or indirectly as a consequence of that selection (Rundle & Nosil, 2005; Schluter, 2000), as in stickleback fish diverging across benthic and limnetic habitats (Hatfield & Schluter, 1999). For flowering plants, biologists have long suggested that interactions with animal pollinators could be the source of the divergent selection and thus a crucial ecological factor in speciation (Grant, 1949). Decades ago, Grant and Grant (1965) and Stebbins (1970) argued that two types of flower phenotypes could specialize on different pollinators (e.g., hummingbirds versus bees). The phenotypes could differ in flower morphology, color, scent, or other traits that influence pollination. A phenotype could affect the attraction of specific pollinators or the effectiveness of that pollinator at transferring pollen due to mechanical fit to the flower (Grant, 1992). We would expect hybrids with intermediate phenotype to receive fewer pollinator visits (or be less effective at pollen transfer), producing divergent selection (or disruptive selection if the two morphologies are sympatric). In this scenario of *pollinator-mediated divergent selection* (sensu Waser & Campbell 2004), hybrids have low pollination success, which represents a form of postzygotic reproductive isolation. Furthermore, because pollinators are agents of gene flow (by moving male gametes) as well as selection, the same set of traits under divergent selection can produce prezygotic reproductive isolation as a byproduct, in the form of assortative mating (Dieckmann & Doebeli, 1999; Rice, 1987). Since angiosperms have diversified contemporaneously with groups of animal pollinators and most species depend at least in part on animal pollination (Ollerton et al., 2011), pollinator-mediated divergent selection is hypothesized to be common.

Ecological speciation in plants could, however, be driven alternatively, or in addition, by adaptation to different environments unrelated to pollinators. For example, two incipient species could show physiological adaptation of vegetative traits to different soil habitats (McNeilly & Antonovics, 1968) or defensive adaptation of leaves to different herbivores (Marquis et al., 2016). In such cases, selection will differ across environments inhabited by two incipient species. For simplicity, we refer to all such ecological mechanisms not involving pollinators as *environment-mediated divergent selection*. Hybrids between two incipient species might have low fitness (postzygotic reproductive isolation) because of genomic incompatibilities (Dobzhansky–Muller incompatibilities; Fishman & Willis, 2001) produced by different alleles providing adaptation to the different environments. Or the hybrids could be poorly adapted specifically to the parental environments by falling between niches (Schluter, 2000).

These mechanisms of ecological speciation can be tested by measuring natural selection on floral traits and vegetative traits in parental and hybrid habitats. The ideal tests would utilize common gardens with hybrids and parental types, so that fitness of individuals with hybrid traits, and how fitness depends on pollination versus other fitness components, can be assessed in particular environments (Rieseberg & Carney, 1998). It is valuable to include advanced generation hybrids, because that increases the range of trait expression, allowing for powerful tests of selection. Pollinator-mediated divergent selection predicts that hybrids will have low pollination success, with selection on floral traits being divergent or disruptive in form. Environment-mediated divergent selection predicts that traits affecting survival (or reproduction unrelated to pollination), such as vegetative traits, will experience divergent selection.

Some studies including hybrid as well as parental phenotypes have characterized the form of selection on floral traits that influence pollination (Campbell et al., 1997; Ippolito et al., 2004; Wesselingh & Arnold, 2000). However, only a few such studies have compared the form of selection on those traits in both parental and hybrid habitats. Hummingbirds preferred red over yellow flowers in all sites of *Mimulus auranticus* while hawkmoths preferred yellow flowers (Streisfeld & Kohn, 2007), leading to divergent selection between coastal and inland sites. For vegetative traits, divergence and selection were measured across taxa of *Protea* (Carlson et al., 2010) and *Clarkia* (Mazer et al., 2022), but studies of selection on leaf traits that include responses of hybrids are also rare (Ludwig et al., 2004). Remarkably few studies have attempted to compare patterns of selection on floral versus vegetative traits across parental and hybrid habitats (but see study of *Clarkia xantiana*; Anderson et al., 2015).

Even when one of these forms of selection has been measured across habitats, its strength has rarely been compared to clines between the species (Stankowski et al., 2015; Streisfeld & Kohn, 2005). A cline is a gradual change in a trait across an environmental gradient. The width of a cline is expected to be proportional to dispersal distance divided by the square root of the difference in selection between ends of the cline (Barton & Hewitt, 1985; Endler, 1977). For a quantitative trait, cline width also scales inversely with the square root of genetic variance (Barton & Gale, 1993). A steeper, narrower cline for a particular phenotypic trait than for putatively neutral loci is often used to infer the action of natural selection (e.g., Brennan et al., 2009; Campitelli & Stinchcombe, 2012; Stankowski et al., 2017). Some neutral processes, including linkage disequilibria between selected and neutral traits due to admixture, can, however, also generate clines (Barton & Shpak, 2000), and the relationship between cline width and selection strength has received little testing (Carvalho et al., 2022). Using contemporary selection to test this relationship requires assuming that ongoing selection reflects historical selection that gave rise to the cline. Previous studies of selection across plant hybrid zones did not address whether differences in selection could explain differences in cline width between floral and vegetative traits.

In this study, we use common gardens including F_2_ hybrids at sites of two parental species of *Ipomopsis* and their natural hybrids to compare patterns of natural selection on floral and vegetative traits that exhibit clines across the hybrid zone (Campbell et al., 1997, 2018). This work builds upon a wealth of background information for this system of closely related species (Porter et al., 2010). Common garden experiments have demonstrated that each species has higher fitness than its congener when measured in its own site of origin (Campbell & Waser, 2007; Campbell et al., 2008). That result indicates some divergent selection, but by itself does not identify traits under selection. Other studies have identified floral traits that influence pollinator visitation or pollen transfer, including corolla length and width, flower color, nectar production, and positions of anthers and stigmas (Campbell, 1989, 1991; Campbell et al., 1991, 1996, 1997; Mitchell, 1993). Some leaf traits, including photosynthetic water-use efficiency, influence survival (Navarro et al., 2022). But with the exception of one study on pollinator visitation (Campbell et al., 1997), those studies did not compare selection across parental habitats, as required to test pollinator-mediated and environment-mediated selection. By measuring selection on those traits and additional vegetative traits suspected to influence fitness, we provide tests of pollinator-mediated divergent selection and environment-mediated divergent selection as competing explanations for ecological speciation. We address the following questions.

Q1. Are floral traits or leaf traits under divergent selection across parental habitats as expected in ecological speciation?
Q2. What is the form of selection on these traits inside the hybrid zone, and does that selection differ from selection in the parental habitats?
Q3. How do floral and vegetative traits compare in the intensity of selection, and does one type of trait contribute more to selection, supporting either pollinator-mediated or environment-mediated divergent selection?
Q4. Is the strength of divergent selection associated with the steepness of the cline in the trait across the natural hybrid zone?

## Methods

### Study system

We employed field common gardens at Poverty Gulch, Gunnison County, CO, USA, where a natural hybrid zone occurs between *Ipomopsis aggregata* ssp. *aggregata* and its congener *Ipomopsis tenuituba* ssp. *tenuituba* (Campbell et al., 1997). Plants of both species are self-incompatible and monocarpic, almost always flowering during only one season before setting seed and then dying (Campbell et al., 2008). Common gardens were established from seed at three sites: the *I. aggregata* site of parental origin (site L in Campbell et al., 1997), the *I. tenuituba* site (site C) and a site at the center of the natural hybrid zone (site I).

At these sites, plants of *Ipomopsis* emerge as seedlings in the spring, and spend 2 to 12+ years as a small rosette of leaves before sending up flowering stalk(s) during their year of flowering (Campbell et al., 2008). Broad-tailed and rufous hummingbirds (*Selasphorus platycercus and Selasphorus rufus*) are the main pollinators at the *I. aggregata* site (Campbell et al., 1997). Hawkmoths (*Hyles lineata*) are rarely seen at that site but are more common at the hybrid and *I. tenuituba* sites (Aldridge & Campbell, 2007; Campbell et al., 1997). In the presence of both hummingbirds and hawkmoths, overall visitation rate was higher to flowers of the two parental species than to hybrids (Campbell et al., 1997). Selection mediated by pollinators may differ between the parental sites, providing a source of divergent selection. The parental sites also differ in water availability that may impose selection particularly on vegetative traits (Navarro et al., 2022). Conditions are warmer during the day, and drier, at the *I. tenuituba* site than at the *I. aggregata* site, despite its higher elevation, because of slope and aspect (Wu & Campbell, 2006).

Previous common garden experiments in the same system addressed the fitness of different classes of plants (parental, F_1_ hybrid, and F_2_ hybrid). These studies demonstrated local adaptation in which the fitness of the home species exceeded that of the away species. In common gardens started in 1994, *I. tenuituba* had fitness of zero in the *I. aggregata* site, and *I. aggregata* had fitness 0.20 as high as *I. tenuituba* at the *I. tenuituba* site (Campbell & Waser, 2007). In a second experiment, *I. tenuituba* had fitness 0.46 times as high as *I. aggregata* in the *I. aggregata* site (Campbell et al., 2008). Hybrids had fitness as high or higher than expected under an additive model of fitness (Campbell et al., 2008). The current study emphasizing F_2_ individuals was designed for measuring selection rather than comparing plants across type of parents.

### Design of common gardens

Common gardens planted for this study starting in 2007 have already been described (Campbell & Powers, 2015; Campbell et al., 2022a), so only an overview is given here. These previous studies used some of the same data to address different questions, including temporal variation in selection on flower traits (Campbell & Powers, 2015) and genetic variation in the traits (Campbell et al., 2022b). Selection on vegetative traits in these common gardens has not yet been addressed.

Seeds consisting of *I. aggregata* (AA), *I. tenuituba* (TT), or hybrids (F_1_ and F_2_) were planted into each site in randomized blocks without removal of any existing vegetation. As there is no seed bank, it was possible to follow the fate of individual seeds planted on a 10 cm × 10 cm grid system. Once a seedling emerged, it was marked with a metal tag to facilitate identification. Plants were followed until they flowered and then died, or died without flowering. In total, 4,512 seeds from 42 full sibships were planted (Supplementary File S1), including 36 sibships that were planted at all three sites in 2007, and an additional 6 sibships of F_2_ individuals planted in 2008 at the *I. aggregata* site. In the summer following seed planting, 666 seedlings established, of which 477 were alive in 2009 when trait measurements began. Vegetative traits were measured on 394 plants, although not every trait was measured on every plant. A total of 192 plants in the common gardens were measured for floral traits and seed production, in 2010–2018. We supplemented the floral trait data set with an additional 71 naturally occurring flowering individuals in the years of 2013–2016 to yield a total sample size of 263 flowering individuals. At the end of the study in 2018, nearly all plants in the common gardens had died, either with or without flowering.

### Measurement of traits and fitness

Selection was characterized by the relationship between traits and fitness using methods of phenotypic selection analysis. The traits were chosen either because previous information suggested they were under selection in at least one of the parental species (all but trichome density; Campbell et al., 1991; Mitchell, 1993; Campbell et al., 1997; Meléndez-Ackerman and Campbell, 1998; Navarro et al., 2022) or exhibited clines across the hybrid zone (all but water-use efficiency which was only measured in three sites; Campbell et al., 2018). We began measuring traits two summers after seed planting, as plants are only small seedlings during the first summer. Vegetative traits measured on rosettes prior to flowering included: specific leaf area (SLA), leaf trichome density, and water-use efficiency (WUE = photosynthetic rate/stomatal conductance) obtained from leaf gas exchange (methods in Campbell et al., 2022b; Wu & Campbell, 2006). Trichome density and SLA were measured in multiple years for each plant, and averaged prior to analysis. We obtained 981 measurements of SLA, 567 of trichome density, and 272 of WUE from 394 vegetative plants. Floral traits included: petal color (reflectance in the red compared to reflectance in the green), corolla length, corolla width, anther insertion (difference between corolla length and length of the longest stamen), and nectar production rate. Measurement of these traits is described in Campbell et al. (2022b) and references therein. The floral traits were measured on multiple flowers per plant and then averaged for each individual prior to analysis. We obtained two to 10 measurements of floral size traits, two to four measurements of flower color, and one to five measurements of nectar production from each of 263 flowering plants.

For vegetative traits (SLA, leaf trichome density, WUE) we used survival to flowering as the fitness component (assuming survival if the plant lived to 2018). Whereas it is theoretically possible that these traits could also influence flower number or seeds per survivor through effects on resource acquisition during earlier parts of the lifecycle, a previous study of *I. aggregata* found no evidence that selection on these traits differed whether flower number was included or not in the fitness estimate (Navarro et al., 2022). For the floral traits, we used number of seeds per flower produced by the plant as the fitness component, as floral traits can only directly influence fitness after flower formation (see methods in Campbell, 1997). Analysis of these two data sets (394 vegetative plants and 263 flowering plants) thus proceeded separately.

### Statistical analysis

#### Q1 Divergent selection across parental habitats

To address divergent selection, we analyzed fitness values from the two parental sites. For floral traits, independent variables in the models were: the factors of site and year, the continuous trait value, and interaction between the trait value and site (see model formulation in Table 1). Year was included as a factor because it accounted for a large portion of the variation in seeds per flower (*R*^2^ = 0.23 in one-way ANOVA, *p* < .0001). Since vegetative traits were averaged across multiple years of measurement for each plant, for those traits, the independent variables were site, the trait value, and their interaction. A significant interaction indicated that directional selection differed across the two parental sites, the signature of divergent selection. If an interaction was detected, we then obtained separate selection gradients for the two parental sites, by running a model with the factor of site (and year in the case of floral traits), and the trait value nested within site. Nesting trait within site generates a separate selection gradient for each site. All trait values were standardized to a mean of 0 and standard deviation of 1 across the set under analysis, and fitness values were relativized by dividing by the mean. As a result, slopes of fitness on traits yielded standardized selection gradients (Kingsolver et al., 2001). Although the main focus was divergence in directional selection, for traits experiencing detectable directional selection, we checked whether quadratic selection (stabilizing or disruptive) also occurred by running models with additional terms of the standardized trait value squared and its interaction with site.

**Table 1.** Analyses of divergent selection across parental habitats. Chi-square values are reported from likelihood ratio statistics for type 3 analysis in a generalized linear model. For the floral trait dataset, the model was specified in procedure Genmod of SAS as: Y = Site + Year + Trait + Site*Trait, where Y is relative seeds per flower, using a normal distribution and identity link. The vegetative trait model was w = Site + Trait + Site*Trait, where w is relative survival to flowering, using a binomial distribution and logit link. Number of plants (*N*) is provided in parentheses

		Source of variation			
Trait	Statistic	Site	Year	Trait	Site*trait
Length (*N* = 172)	χ^2^	2.17	**44.00**	0.02	**8.63**
	*p*	.1405	**<.0001**	.8885	**.0033**
Width (*N* = 172)	χ^2^	1.01	**41.36**	2.11	1.32
	*p*	.3159	**<.0001**	.1466	.2500
Anther insertion (*N* = 172)	χ^2^	1.34	**34.09**	0.83	**13.23**
	*p*	.2472	**<.0001**	.3623	**.0003**
Color (*N* = 131)	χ^2^	0.06	**49.91**	**5.70**	0.12
	*p*	.8095	**<.0001**	**.0170**	.7280
Nectar production (*N* = 125)	χ^2^	0.00	**41.83**	0.20	0.22
	*p*	.9924	**<.0001**	.6517	.6360
SLA (*N* = 273)	χ^2^	**14.46**	NA	**13.78**	1.57
	*p*	**.0001**	NA	**.0002**	.2096
Trichome density (*N* = 206)	χ^2^	1.87	NA	0.95	0.15
	*p*	.1710	NA	.3303	.7031
WUE (*N* = 172)	χ^2^	**16.42**	NA	1.27	1.57
	*p*	**.0001**	NA	.2606	.2102

Bold font indicates p < 0.05.

Traits were standardized to the mean across the entire data set (global scaling as defined by De Lisle & Svensson, 2017) because the distribution of phenotypes at the start of the experiment was fixed and constant across sites. We emphasize results based on global scaling for fitness because the vegetative traits are likely under hard selection and the floral traits experience selection by pollinators that make choices among sites as well as within them (Campbell et al., 1997), resulting in some gene flow across the hybrid zone. We, however, also present results from local scaling of fitness (by the mean specific to a population) for comparison.

For all of these univariate selection analyses, generalized linear models were implemented using procedure Genmod in SAS ver. 9.3 using a normal distribution and identity link for the response variable of relative seeds per flower and a binomial distribution and logit link for relative survival to flower (global scaling only because a binary response is required). Use of a binomial distribution is most appropriate for testing statistical significance of a binary response variable like survival, but for direct comparison of intensities of selection across the two types of traits, we also ran the models using a normal distribution and identity link.

In addition to these univariate estimates of selection, we performed multivariate selection analysis to account for the influence of correlated traits (see correlations in Supplementary File S2). For floral traits, we analyzed relative seeds per flower using the factor of site and each trait value nested within site. Nesting the continuous effect of each floral trait within site produces separate selection gradients at each of the two parental sites. The vegetative data set had a particularly large number of missing values due to logistical constraints imposed on hiking equipment for measuring gas exchange into these remote sites. So for vegetative traits, we used multiple imputation methods to infer missing data. We performed partial imputation of the data set 40 times (recommended replication following Graham et al., 2007), using procedure MI in SAS and a model with relative survival and the three standardized traits (Newman, 2014). To estimate directional selection at each site, we then ran multiple regression of relative fitness on the three traits for each imputation and combined the partial imputations using procedure MIANALYZE.

#### Q2 Selection at the hybrid site

Selection at the hybrid site could take a variety of forms, including disruptive selection due to genomic incompatibilities or the presence of both pollinators or habitat types, or stabilizing selection in the case of bounded hybrid superiority (Arnold, 1997). So at the hybrid site garden, we first ran univariate models containing the independent variables of year (for flower traits only), the standardized trait value, and the squared standardized trait value to allow for nonlinear selection. If there was no detectable effect of the squared trait value, we then ran a model without that quadratic term to check for linear selection.

#### Q3 Comparison of strength of selection

We used the directional standardized selection gradients to compare the intensity of selection on vegetative versus floral traits. To compare specifically the importance of divergent selection, we calculated the difference between the standardized selection gradients in the two parental habitats in the direction that would explain the observed differences in trait means. For example, if selection favored a higher trait value in the *I. tenuituba* site, then divergent selection was positive if the mean trait value was also higher at that site, but negative if the trait value was lower at that site. Thus positive values indicated concordance of selection with the observed traits means. These differences were compared across the two classes of traits. Comparisons were performed both with univariate selection gradients and with multivariate gradients.

#### Q4 Association with clines

Clines in all traits except WUE were previously measured in the hybrid zone in 2015–2016 (Campbell et al., 2018). Sigmoidal no-tails clines (Derryberry et al., 2014) were fit to each trait across 12 populations spanning 1.6 km between the highest and lowest populations (Supplementary File S3). Traits had clines differing in steepness, with the order from widest (1.5 km) to narrowest (i.e., steepest; 0.3 km) of corolla width, SLA, nectar production, anther insertion, leaf trichomes, corolla length, and petal color. Thus, we predicted the weakest divergent selection for corolla width and strongest divergent selection for petal color. The prediction was tested by determining the correlation coefficient between cline width and the difference between the standardized selection gradients in the two parental habitats in the direction that would explain the difference in trait means across the cline. The correlation was calculated using the univariate selection measures and also using the multivariate selection gradients.

## Results

### Q1: Divergent selection across parental habitats

**Figure 1.**
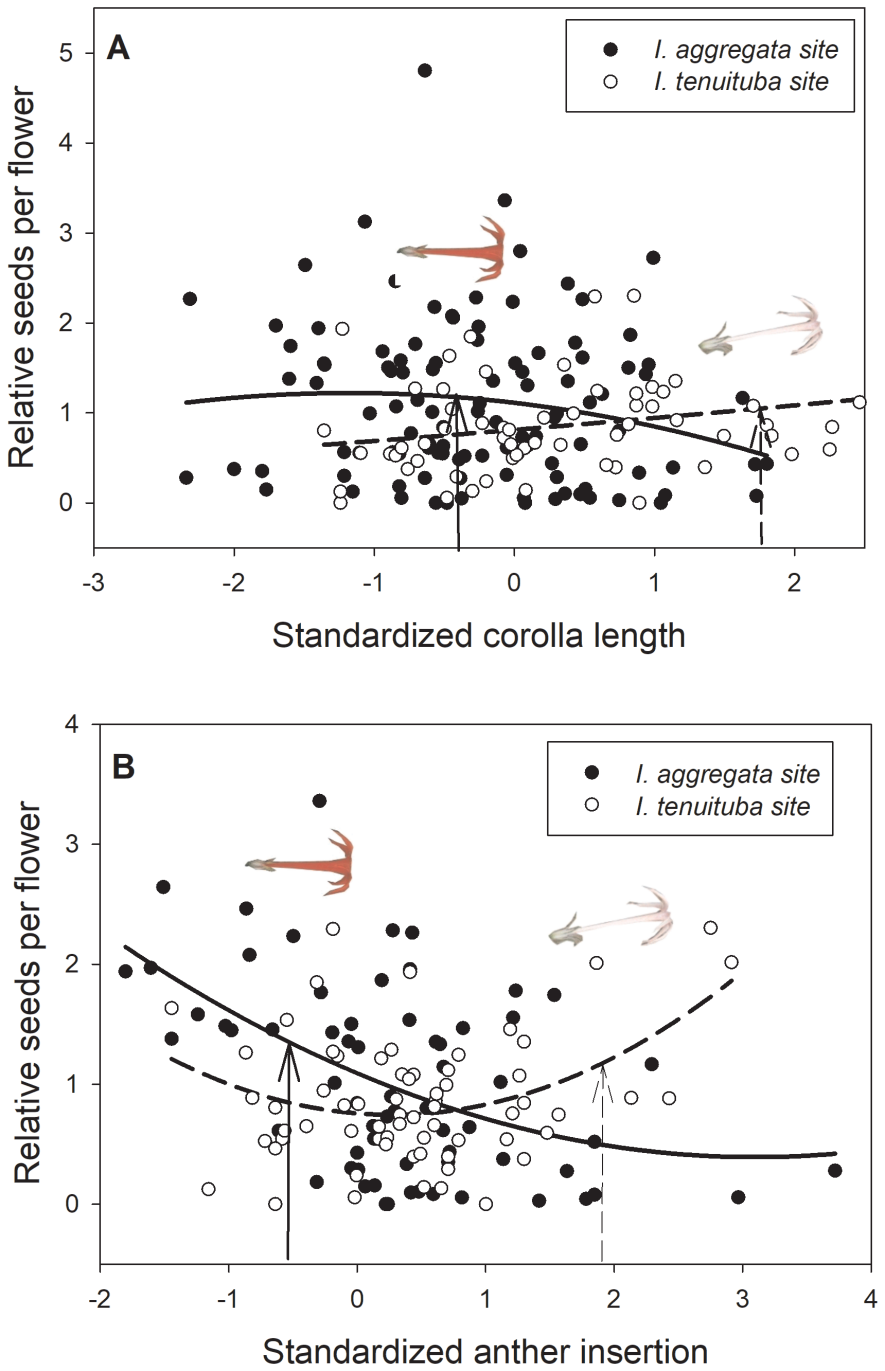
Divergent selection on two floral traits in *Ipomopsis.* Relative seeds per flower as a function of (A) standardized corolla length and (B) standardized anther insertion in the two parental sites. Selection differed significantly across sites (*p* < .01 for site × trait interaction; Table 1). The fitted curves show best-fitting quadratic regressions. Corolla length averaged 27.0 mm for *I. aggregata* in the *I. aggregata* site (standardized value = −.42; solid arrow) and 35.3 mm for *I. tenuituba* in the *I. tenuituba* site (standardized value = 1.74; dashed arrow). Anther insertion averaged 0.75 mm for *I. aggregata* in the *I. aggregata* site (standardized value = −0.66; solid arrow) and 4.92 mm for *I. tenuituba* in the *I. tenuituba* site (standardized value = 1.58; dashed arrow). Inset photos show single flowers of the two species positioned above the mean values at their home sites.

**Figure 2.**
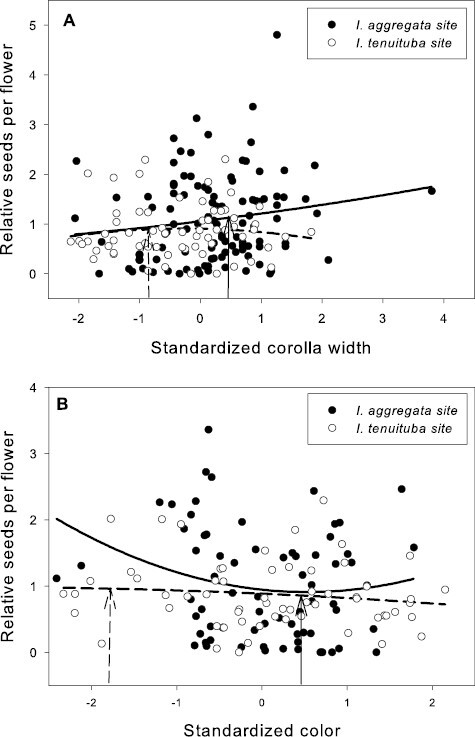
Relative seeds per flower as a function of (A) standardized corolla width and (B) flower color in the two parental sites. Neither trait showed evidence of divergent selection across the two sites. Selection favored wider corollas at the *I. aggregata* site (*p* < .05) and was not significant at the *I. tenuituba* site. Selection overall favored less red flowers (*p* < .05, Table 3). The fitted curves show best-fitting quadratic regressions. Corolla width averaged 3.52 mm for *I. aggregata* in the *I. aggregata* site (standardized value = 0.46; solid arrow) and 3.06 mm for *I. tenuituba* in the *I. tenuituba* site (standardized value = −0.85; dashed arrow). Relative reflectance in the red compared to the green averaged 0.61 for *I. aggregata* in the *I. aggregata* site (standardized value = 0.59; solid arrow) and 0.14 for *I. tenuituba* in the *I. tenuiutuba* site (standardized value = −1.80; dashed arrow).

Basic demographic results are in Supplementary File S4. Selection estimates were very similar between global and local scaling of fitness (Supplementary File S5). For simplicity, we present results from global scaling. Of the five measured floral traits, two traits (corolla length and anther insertion) showed evidence of divergent selection, as indicated by a significant trait by site interaction on the fitness measure of relative seeds per flower (Table 1). Nesting the effect of corolla length within a site, longer corollas were favored at the *I. tenuituba* parental site (β = 0.17 ± 0.08 SE) and shorter corollas at the *I. aggregata* parental site (β = −0.15 ± 0.07, *p* = .0132), which accords with the natural difference in corolla length (Figure 1A). Selection on that trait was largely directional, as a model with the additional quadratic effect of length nested within site detected no significant quadratic term (*p* = .8659). More strongly inserted anthers were favored at the *I. tenuituba* site (β = 0.16 ± 0.10) and less inserted anthers at the *I. aggregata* site (β = −0.27 ± 0.08; *p* = .0003 for overall effect of anther insertion nested within site), which also accorded with differences between the species in nature (Figure 1B). Anther insertion also had a quadratic effect on relative seeds per flower (*p* = .0263 in model with site, year, trait nested within site, and trait^2^ nested within site). Thus selection was disruptive as well as directional in form, but the most highly inserted anthers naturally occurring still yielded highest fitness at the *I. tenuituba* site and highly exserted anthers (negative values) yielded highest fitness at the *I. aggregata* site (Figure 1B). In contrast, corolla width, petal color, and nectar production showed no evidence of divergent selection (site by trait interaction *p* > .05, Table 1). Overall, selection favored flowers that were paler in color across sites (lower values for the measure of redness, *p* = .0170; Table 1; Figure 2B) and wider flowers at the *I. aggregata* site (β = 0.15 ± 0.07, *p* = .0415 in a model with effect of width nested within site; Figure 2A). No directional selection on nectar production was detected. Selection gradients from a multivariate model with all traits nested within site gave qualitatively similar results, with the exception of length and color at one site each (Table 3).

**Table 3.** Selection gradients at parental sites compared with cline widths. Estimates of selection (β) were obtained from models for each trait with the factor of site and the trait value nested within site: Y = Site + Trait(Site) in procedure Genmod of SAS. For floral traits, year of blooming was also included as a factor. For vegetative traits, statistical significance of univariate β came from a model with a binomial distribution (parameter estimates in parentheses), but parameter estimates from a model with a normal distribution and identity link are provided for direct comparison with selection on floral traits. Multivariate estimates of β for vegetative traits required multiple imputation methods under the assumption of multivariate normality, and separate models were run for each site. Concordant difference in selection refers to the difference between β in the two sites in the direction that matches the difference in average observed trait values. Values for cline width come from a previous study that fit sigmoidal clines across 12 populations (Campbell et al., 2018). Percentage evolvability was measured in common gardens in the field by (Campbell et al., 2022b), except for the trait of anther insertion which is newly reported based on applying these methods to the absolute value of anther insertion (Opedal et al., 2017).

Trait	Univariate β at *I. agg* site	Univariate β at *I. ten* site	Concordant difference in univariate β	Multivariate β at *I. agg* site	Multivariate β at *I. ten* site	Concordant difference in multivariate β	Cline width (km)	Evolvability (%)
Length	−0.153*	0.168*	0.321**	−0.023	0.242	0.265	0.45	0.70
Width	0.151*	0.016	0.135	0.209*	−0.087	0.296*	1.51	0.49
Anther insertion	−0.275***	0.159	0.434***	−0.297***	−0.138	0.159	0.77	3.34
Color	−0.112	−0.151*	0.039	−0.250*	0.031	−0.281*	0.33	6.32
Nectar	−0.000	−0.054	0.054	−0.028	0.053	−0.081	0.78	0.00
SLA	(−0.686**) −0.332***	(−0.328) −0.186	(−0.358) −0.146	−0.322**	−0.244*	−0.078	1.35	0.52
Trichome density	(0.200) 0.114	(0.087) 0.047	(−0.113) −0.067	0.085	0.071	−0.014	0.56	3.15
WUE	(−0.035) −0.021	(0.753) 0.160	(0.786) 0.162	−0.026	0.199	0.225	NA	0.26

*p* < .05 for selection gradient. **p < .01. ***p < .001.

**Figure 3.**
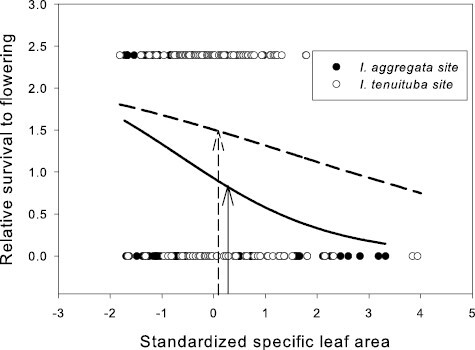
Relative survival to flowering as a function of standardized specific leaf area (SLA) in the two parental sites. Selection was directional, favoring lower SLA (*p* < 0.001; Table 1). Curves show logistic fits separately by site. SLA averaged 186.7 cm^2^/g for *I. aggregata* in the *I. aggregata* site (standardized value = 0.24; solid arrow) and 180.9 cm^2^/g for *I. tenuituba* in the *I. tenuituba* site (standardized value = 0.08; dashed arrow) in this data set. Species differences across the hybrid zone were more extreme in a previous study (180 cm^2^/g and 150 cm^2^/g in 2015–2016 at the *I. aggregata* and *I. tenuituba* ends of the hybrid zone; Campbell et al., 2018).

None of the vegetative traits showed evidence of divergent selection, as no site by trait interactions on fitness were detected (Table 1). Selection favored lower SLA (i.e., thicker leaves) at both parental sites (*p* = .0003 in a model with the effect of SLA nested within site; Figure 3). No directional selection was detected on trichome density or photosynthetic water-use efficiency at either parental site. Within site phenotypic correlations between these traits were weak (*r* ranged from −0.19 to 0.24, all *p* > .05; Supplementary File S2).

### Q2: Selection in the hybrid zone

No quadratic selection was detected for any trait within the hybrid site (Table 2), and so we estimated linear selection gradients from models without a quadratic term. Directional selection acted on two traits. Selection favored shorter corollas within the hybrid site (β = −0.24 for corolla length, *p* = .0062 in a model that also included the factor of year. In addition, selection favored lower values of SLA (β = −0.42 using normal distribution, *p* = .0007). Both of those traits experienced selection similar to that observed at the *I. aggregata* site (β = −0.15 and −0.33, respectively, Table 3, Supplementary File S6).

**Table 2.** Analysis of selection at the hybrid site. Statistics for the effects of year and trait are for models without the quadratic term: Y = Year + Trait, where Y = relative seeds per flower for floral traits and relative survival to flowering for vegetative traits. Statistics for Trait^2^ are from the full model: Y = Year + Trait + Trait^2^. Chi-square values are reported from likelihood ratio statistics for type 3 analysis in a generalized linear model. Number of plants (*N*) is provided in parentheses.

Trait	Statistic	Year	Trait	Trait^2^
Length (*N* = 64)	χ^2^	**44.78**	**7.09**	0.12
	*p*	**<.0001**	**.0077**	.7245
Width (*N* = 64)	χ^2^	**41.74**	0.47	0.54
	*p*	**<.0001**	.4929	.4641
Anther insertion (*N* = 63)	χ^2^	**40.40**	2.84	0.47
	*p*	**<.0001**	.0921	.4932
Color (*N* = 44)	χ^2^	**25.09**	0.91	1.24
	*p*	**.0001**	.3393	.2664
Nectar production (*N* = 36)	χ^2^	**20.15**	2.49	1.11
	*p*	**.0012**	.1144	.2923
SLA (*N* = 110)	χ^2^	NA	**11.52**	0.24
	*p*	NA	**.0007**	.6250
Trichome density (*N* = 71)	χ^2^	NA	3.32	1.03
	*p*	NA	.0686	.3110
WUE (*N* = 67)	χ^2^	NA	0.40	0.70
	*p*	NA	.5279	.4043

Bold font indicates *p* < .05.

### Q3: Comparison of strength of selection

Using the lifecycle components of fitness that were most appropriate for the given trait (seeds per flower for floral traits and survival to flowering for vegetative traits), four out of five floral traits, and one of three vegetative traits, were under selection at one or both sites (Table 3). The absolute value of the univariate selection gradient averaged 0.12 for floral traits and 0.14 for vegetative traits (using a normal distribution) across both parental sites. Only floral traits showed any evidence, however, of divergent selection across the two parental habitats. The difference in the univariate β between parental habitats in the direction matching the cline was always positive as expected and averaged 0.21 for floral traits, although it was non concordant for flower color using the multivariate estimates (Table 3), largely due to selection for paler flowers in the *I. aggregata* site. The difference in β between parental habitats was negative and not concordant for two of three vegetative traits (Table 3), thus tending to counteract the observed cline in those cases.

### Q4: Association with clines

There was little correspondence between the strength of divergent selection (difference in β in Table 3) and the width of the cline. Although theory predicts a negative correlation, the actual correlation coefficient across seven traits was −0.23 using univariate and 0.34 using multivariate estimates of β, neither significantly different from zero (*p* > .45). The narrowest cline was for flower color, and so should have corresponded with intense divergent selection, and yet selection actually favored paler flowers similarly in the two parental sites (Table 3).

## Discussion

### Ecological speciation: Q1–Q3

Pollinator-mediated selection and environment-mediated selection offer two nonmutually exclusive alternatives for the divergent natural selection that drives ecological speciation in flowering plants. Pollinator-mediated selection acts on floral traits, whereas environment-mediated selection can act on vegetative traits. Although pollinator-mediated selection is frequently demonstrated, it is rare to include the geographic element that tests for divergent selection due to different pollinators in sites occupied by the two parental species (Van der Niet et al., 2014). Here we uncovered comprehensive evidence for divergent selection due to pollinators on some floral traits.

As in previous studies (Campbell & Waser, 2007; Campbell et al., 2008), each species of *Ipomopsis* had lower fitness than the home species if transplanted to a new site (Supplementary File S4), although that signature of local adaptation was not as pronounced as in previous studies. This finding of local adaptation suggests some traits would experience different patterns of selection at the two sites. The current study revealed divergent selection across the parental habitats on two floral traits while finding no evidence for divergent selection on vegetative traits that differed between the two species of *Ipomopsis*. These results suggest pollinator-mediated divergent selection is the main driver of ecological speciation in this system (Q1), although predispersal seed predation could also affect seed production (Campbell et al., 2022a). The difference in divergent selection between types of traits was not an artifact of statistical power, as sample sizes were larger for vegetative traits, and directional selection was detected on specific leaf area. Selection on the floral traits within a site of natural hybrids was similar to that of selection at the *I. aggregata* site (Q2), consistent with the demonstrated hummingbird preference for *I. aggregata* over hybrids and *I. tenuituba* plants placed into the center of the hybrid zone (Campbell et al., 1997).

A major caveat is that only female fitness was measured here. On average across a population fitness should equal the average of female fitness and male fitness, but it is well known that trait effects on male fitness can depart considerably from those on female fitness (Christopher et al., 2020; Stanton et al., 1986). In *I. aggregata*, variance in female fitness, based on total seed production, was much larger than variance in male fitness, based on seeds sired (Campbell, 1998), suggesting less opportunity for selection through male function and the possibility that we have here overestimated selection. What matters more for testing a correlation with cline width is, however, whether that bias varies across traits. That variation is likely, as only some traits (e.g., corolla width but not corolla length) show differential effects on male versus female pollination success (Campbell, 1989). Corolla width more strongly influences pollen export than pollen receipt since it not only influences visit rate of hummingbirds but also how much pollen a hummingbird removes per visit (Campbell et al., 1996). Anther insertion is another trait likely to have its most direct impact on fitness through male function, as *I. aggregata* plants with less inserted anthers have more pollen removed in a hummingbird visit (Campbell et al., 1998). Its observed effect on seed production could be caused by interference of the position of the anthers with deposition of outcross pollen onto the stigma in this self-incompatible plant. In sum, any difference between traits in the relative importance for male versus female fitness could have contributed to a poor correlation between the estimated selection intensity and cline width.

A second caveat is that selection analysis was not performed for one other floral trait that differs between the two plant species: emission of the volatile organic compound indole from the flowers (Bischoff et al., 2014) which induces hawkmoths to visit flowers (Bischoff et al., 2015).

Despite divergent selection on two floral traits, some floral traits that differ between the species (corolla width, flower color, and nectar production) did not show signatures of divergent selection. The inconsistency may reflect the general paucity of hawkmoths in this region, which means that visitation can be dominated by hummingbirds at all sites (Aldridge & Campbell, 2007). Given the importance of the reward to the pollinators, it is initially surprising to see no directional selection for greater nectar production. In other studies of *Ipomopsis* nectar production has influenced pollinator visitation and pollen export but also has had little effect on seed production (Mitchell, 1993), which might reflect a tradeoff with the greater water requirement to produce more nectar (Powers et al., 2022).

Strong selection was detected on one vegetative trait: specific leaf area. That selection was strongly directional for thicker leaves (lower SLA) than are observed on average at either parental site (Figure 3). Globally, low SLA is associated with drier conditions, as the lower surface area reduces water loss during photosynthesis (Poorter et al., 2009). In a previous experimental study of *I. aggregata*, lower SLA was favored when snowpack was forced to melt earlier than normal in the spring, which causes an extended period of drought prior to summer monsoon rains (Navarro et al., 2022). In the current study, selection on SLA appeared at least as strong at the *I. aggregata* as the *I. tenuituba* site, even though the latter site is drier. That result contrasts with a study of *Boechera stricta*, also in the Colorado Rocky Mountains, that found divergent selection across elevation on SLA. In that study selection favored reduced SLA at a lower elevation (2,891 m), as in our study, but an intermediate SLA at higher elevation of 3,133 m (Wadgymar et al., 2017). In *Ipomopsis*, divergent selection in the direction of the cline was overall stronger for floral traits than for vegetative traits (Q3).

These results are largely consistent with previous research in *Ipomopsis* that has shown effects of floral traits on pollinator visitation and pollen transfer, but there are some discrepancies (see details and explanation of multivariate selection favoring paler flowers in Supplementary File S7), which may result from temporal variation in selection. For example, selection on corolla length has declined in intensity with the recent trend towards earlier snowmelt (Campbell & Powers, 2015; Powers et al., 2022).

### Comparison with other study systems

Few other plant systems have utilized transplants to compare divergent selection on vegetative versus reproductive traits across an environmental gradient, and results have been mixed. One such study examined *Boechera stricta* across an elevational gradient, but because that species is self-fertilizing, pollinator-mediated selection on traits of individual flowers was not considered (Wadgymar et al., 2017). A study of *Clarkia xantiana*, which is insect-pollinated, found strong spatial differences in selection that were largely concordant with expressed trait differences across populations, but included only one floral trait (Anderson et al., 2015). Other than *Ipomopsis*, two of the best-studied systems of divergent selection involving pollinators are those of *Aquilegia* and *Mimulus aurantiacus*. The European species of *Aquilegia* are less differentiated by pollinator type, and in that system selection on vegetative traits was generally stronger than on floral traits (Castellanos et al., 2011), in contrast with the strong pollinator specialization of North American species (Fulton & Hodges, 1999), and for some vegetative traits was divergent in a way that could explain population differences (Alcantara et al., 2010). In *Mimulus aurantiacus*, the hummingbirds that are more common near the coast favor more red flowers while inland hawkmoths favor more yellow flowers (Streisfeld & Kohn, 2007), consistent with divergent selection mediated by pollinators. However, some ecophysiological traits also show clinal variation and experience selection concordant with the trait variation (Sobel et al., 2019). Our finding of stronger divergent selection on floral than vegetative traits in *Ipomopsis* adds to the literature showing that different pollinators can exert selection on floral traits in different directions. By demonstrating contemporary divergent selection, the results are consistent with the long-held contention that pollinators have driven diversification of flower traits in the family Polemoniaceae (Grant & Grant, 1965).

### Association with clines: Q4

Our study allowed testing of how well divergent selection explains the width of clines across a hybrid zone. Although some floral traits in *Ipomopsis* experienced divergent selection, the relative strength of divergent selection did not closely match the relative steepness of the cline, as is often assumed. Cline width is expected to decrease (steeper cline) with stronger divergent selection, but a significant correlation across traits was not detected. Steeper clines are also expected when genetic variance, relative to the difference in trait means between ends of the cline, is higher (Barton & Gale, 1993). That prediction can be tested, as genetic variance and narrow sense heritability were measured in these same common gardens in the field, by using crosses to form sibships planted as seed and followed through the lifecycle (Campbell et al., 2022b). We standardized genetic variance as evolvability: genetic variance divided by the squared mean of the trait (Opedal, 2019). Evolvability was measured for six of the seven traits with measured clines (Table 3) from these common gardens and others at the same sites (Campbell et al., 2022b). Evolvability of anther insertion was not previously reported, but can be calculated from that same experiment (Table 3). As anther insertion can take on negative and positive values, we calculated evolvability on its absolute value (ratio-scale evolvability; Opedal et al., 2017). Adding that value, we examined the correlation between evolvability and cline width to test the prediction of a negative correlation. The observed correlation was −0.61 (*p* = .1439, *N* = 7 traits). While not a significant correlation, evolvability appeared to do a better job predicting cline width than did the strength of divergent selection, which correlated at the *r* = −0.23 level using univariate estimates. That evolvability was a better predictor is similar to the general finding that evolvability is positively associated with the extent of trait divergence between plant populations (Opedal et al., 2023).

## Conclusions

Few studies have examined the strength of divergent selection on both reproductive and juvenile traits in the same system, whether in plants or animals (Anderson et al., 2015), and we are unaware of others that also compared both kinds of selection measures with clines. Results for *Ipomopsis* point to a greater role for pollinator-mediated selection than environment-mediated selection in maintaining current species differences, and suggest that only some phenotypic differences between the taxa are currently strongly adaptive. Future studies should continue to test the extent to which divergent selection across parental habitats explains the steepness of clines.

## Supplementary Material

qrad060_suppl_Supplementary_Material

## Data Availability

Data and code are deposited in the Dryad Digital Repository: https://doi.org/10.5061/dryad.79cnp5j2r (Campbell et al., 2023).
