# Supplementary material for: Pollinator and habitat-mediated selection as potential contributors to ecological speciation in two closely related species
Source: Evol Lett. 2023 Nov 23;8(2):311–21. doi: 10.1093/evlett/qrad060 (PMC10959478; doi:10.1093/evlett/qrad060)
Supplement: qrad060_suppl_Supplementary_Material [file qrad060_suppl_supplementary_material.pdf]

Supplementary file S1:

Sample sizes and two fitness components in the common gardens: (1) proportion survived to flowering used for analyzing selection on vegetative traits, and (2) seeds per flower used for selection on floral traits. Seeds per flower per seed planted is a multiplicative combination of those two components. Naturally occurring individuals were added to the data set for measuring selection in 2013-2016.

Site	Type of plant	Seeds planted	Vegetative plants measured	Flowering plants measured	Proportion survived to flowering	Seeds per flower	Seeds per flower per seed planted
I. aggregata	AA	248	16	7	0.022	1.30	0.027
	F1	378	27	10	0.017	0.73	0.013
	F2	1350	96	54	0.017	1.66	0.030
	TT	207	9	1	0.005	3.25	0.015
	Natural *I. aggregata*	NA	NA	47	NA	1.71	NA
Hybrid	AA	240	28	13	0.062	1.14	0.069
	F1	295	30	19	0.056	0.94	0.045
	F2	480	44	13	0.032	1.29	0.040
	TT	139	13	3	0.052	1.31	0.081
	Natural hybrid	NA	NA	24	NA	0.55	NA
I. tenuituba	AA	240	21	7	0.034	1.32	0.039
	F1	301	42	26	0.07	1.31	0.088
	F2	475	46	27	0.077	1.00	0.090
	TT	159	20	12	0.06	1.70	0.095
TOTAL	all	4512	394	263			

Supplementary file S2.
Pearson correlations (A) between floral traits and (B) between vegetative traits. Correlations were calculated separately at each of the two parental sites.

(A) Floral traits

Site	Floral trait	Length	Width	Anther insertion	Color	Nectar production
I. aggregata	Length	**1**	-.01	**0.33*** **	-0.18	-0.11
	Width		**1**	**-0.31*** **	0.10	0.15
	Anther insertion			**1**	-0.21	-0.18
	Color				**1**	**0.32***
I. tenuituba	Length	**1**	0.19	**0.63**** **	**-0.49**** **	**-0.45*** **
	Width		**1**	-0.06	**0.29***	0.19
	Anther insertion			**1**	**-0.42*** **	**-0.49**** **
	Color				**1**	**0.44*** **

(B) Vegetative traits

Site	Vegetative trait	SLA	Trichome density	WUE
I. aggregata	SLA	**1**	-0.19	0.05
	Trichome density		**1**	0.11
I. tenuituba	SLA	**1**	0.11	0.24
	Trichome density		**1**	-0.01

* P < 0.05. *** P < 0.001. **** P < 0.0001

Supplementary file S3:

Clines across the hybrid zone in floral traits measured in 2015 and 2016. Figure is adapted from Campbell et al. (2018). Clines were also measured in 1991 to 1992 (shown in Campbell et al. 2018). For each of 12 populations, Campbell et al. (2018) determined the mean trait value and then standardized it between zero and one by subtracting the minimum across the 12 populations and then dividing by the difference between the maximum and the minimum. Clines were fit to a no-tails model:

$$Y = a + \frac{1}{1+e^{-4\frac{(X-c)}{w}}}$$

where Y = standardized trait value, X = distance, a = intercept, c = center of cline, and w = width of cline (Derryberry et al., 2014). T, H, and A indicate the positions of the *I. tenuituba,* hybrid, and *I. aggregata* common gardens in the current study. In this figure, the narrowest (steepest) cline is for redness, leading us to predict the strongest divergent selection on that trait.

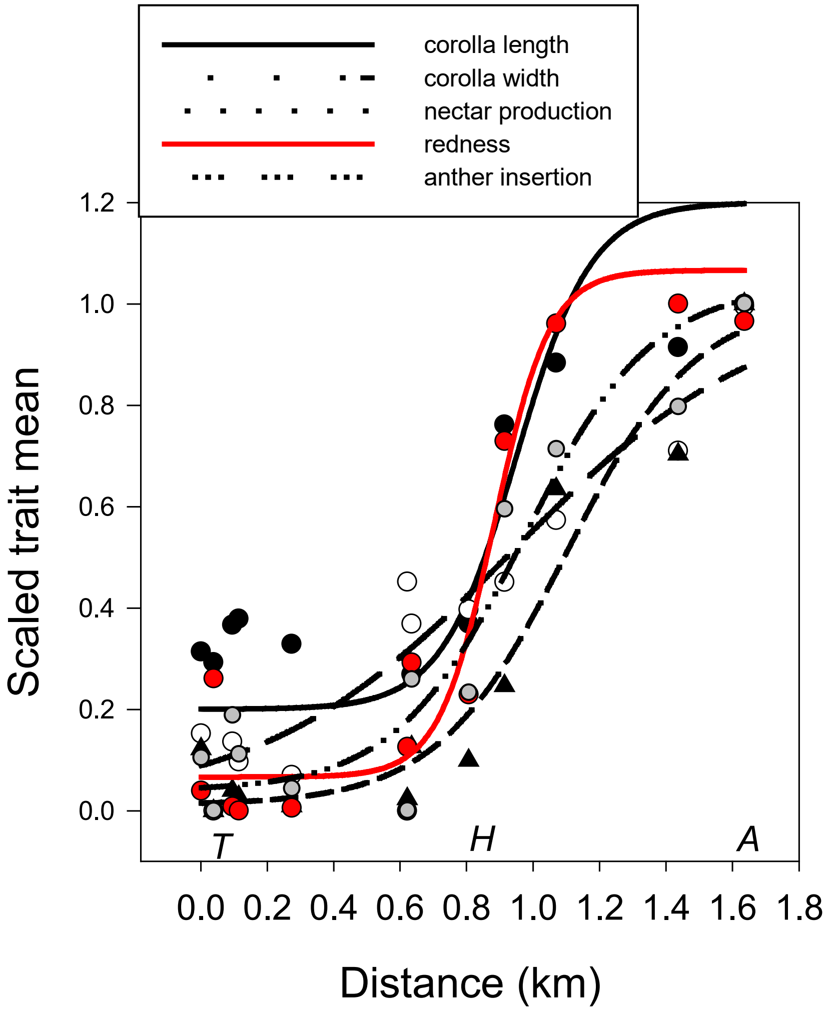

Supplementary file S4:

Basic demographic results

Overall, 4% of the seeds planted with known genetic background survived to flower. As in previous common gardens, *I. tenuituba* had low fitness compared to the home species at the *I. aggregata* site (0.55 times as high for the fitness measure obtained by multiplying survival times seeds per flower; File S1) and, similarly, *I.* aggregata had low fitness compared to the home species at the *I. tenuituba* site (0.42 times as high).

Supplementary file S5

Comparison of univariate selection gradients using global scaling versus local scaling of fitness to its mean value. Estimates of selection (β) were obtained from models for each trait with the factor of site and the trait value nested within site. For floral traits, year of blooming was also included as a factor in the model. For vegetative traits, statistical significance of univariate β came from a model of absolute fitness with a binomial distribution, but parameter estimates from a model of scaled fitness with a normal distribution and identity link for direct comparison with selection on floral traits. Concordant difference in selection refers to the difference between β in the two sites in the direction that matches the difference in average observed trait values. Statistical significance of the difference in selection was assessed by the site by trait interaction in a model Y = Site + Year + Trait + Site*Trait, where Y is relative fitness, using a normal distribution and identity link, and the factor Year was only included for floral traits.

Trait	Univariate β at *I. agg* site		Univariate β at *I. ten* site		Concordant difference in univariate β	
	Global scaling	Local scaling	Global scaling	Local scaling	Global scaling	Local scaling
Length	**-0.153***	-0.141	**0.168***	**0.192****	**0.321****	**0.333****
Width	**0.151***	**0.143***	0.016	0.015	0.135	0.128
Anther insertion	**-0.275*****	**-0.253*****	0.159	0.181	**0.434*****	**0.434*****
Color	-0.112	-0.110	**-0.151***	**-0.167***	0.039	0.057
Nectar	-0.000	0.001	-0.054	-0.071	0.054	0.072
SLA	**-0.332****	**-0.366******	-0.186	-0.171	-0.146	-0.195
Trichome density	0.114	0.126	0.047	0.043	-0.067	-0.083
WUE	-0.021	-0.023	0.160	0.147	0.162	0.170

* P < 0.05. ** P < 0.01, *** P < 0.001. **** P < 0.0001

Supplementary file S6:

Selection on two traits in the hybrid site for comparison with selection observed at the *Ipomopsis aggregata* site. A: Relative seeds per flower as a function of standardized corolla length. The fitted curve shows the best-fitting quadratic regression (compare with closed circles and solid line in Fig. 1A). Seeds per flower declined significantly with corolla length in a model that also accounted for year of flowering, shown by fill color ($P < 0.01$; Table 2). B: Relative survival to flowering as a function of standardized specific leaf area (compare with closed circles and solid line in Fig. 2). Survival to flowering declined significantly with specific leaf area ($P < 0.001$; Table 2).

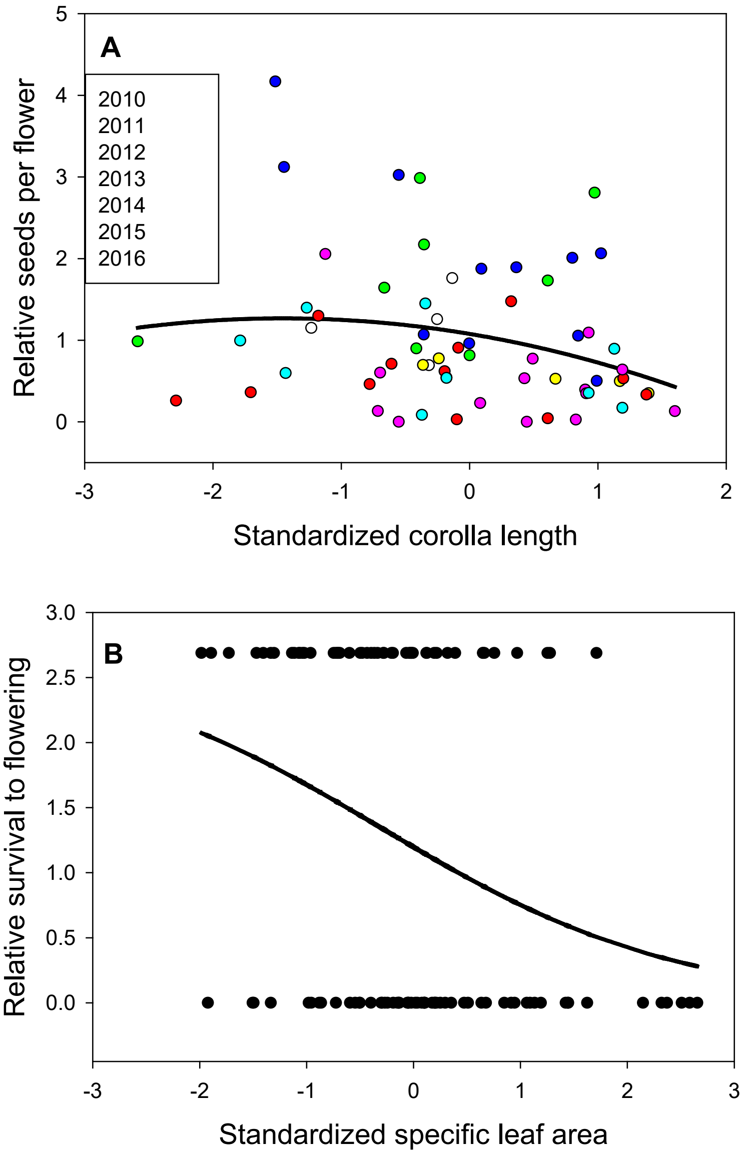

Supplementary File S7.

Comparison of results with previous studies of this system

The current study demonstrated selection in the parental habitats of *Ipomopsis aggregata* and *I. tenuituba.* Here we assess the extent to which these results are concordant with previous studies. In this study system, plants with longer corollas sometimes receive more hummingbird visits (Campbell et al. 1991), and hawkmoths consistently prefer to visit the longer-tubed *I. tenuituba* over *I. aggregata* (Aldridge and Campbell 2007). Effects on pollinator visitation rate do translate into effects on seed production in *Ipomopsis aggregata* and *I. tenuituba*, as seed production is pollen-limited (Campbell and Halama 1993; Campbell et al. 2002; Campbell et al. 2022c). It is thus not surprising that the current study detected selection for longer corollas at the *I. tenuituba* site, although it is less clear why selection favored shorter corollas at the *I. aggregata* site. The observed selection favoring wide corollas at the *I. aggregata* site is reinforced by an independent study that demonstrated hummingbird preference favoring wider corollas at that site (Campbell et al. 1997). The observed selection for less red flowers may result from visitation preference of hawkmoths, as observed with manipulations of flower color under low light levels (Bischoff et al. 2015). Hummingbirds show spontaneous preference for red flowers in this system (Meléndez-Ackerman and Campbell 1998), but despite that previous studies have also revealed selection for the combination of wide and pale flowers (Campbell 2009). As in the few other systems for which selection has been characterized for many decades (e.g. Darwin's finches; Grant and Grant 2002), selection has varied across years. Some of that is systematic; for example, selection on corolla length has declined in intensity with the recent trend towards earlier snowmelt (Campbell and Powers 2015; Powers et al. 2022), likely because seed production has become more water-limited. Other variation is due to idiosyncratic variation in pollinator behavior, for example temporal variation in hummingbird response to corolla length (Campbell et al. 1991; Campbell et al. 1997).

Literature Cited

Aldridge, G. and D. R. Campbell. 2007. Variation in pollinator preference between two *Ipomopsis* contact sites that differ in hybridization rate. Evolution 61:99-110.Bischoff, M., R. A. Raguso, A. Jürgens, and D. R. Campbell. 2015. Context-dependent reproductive isolation mediated by floral scent and color. Evolution 69:1-13.

Campbell, D. R. 2009. Using phenotypic manipulations to study multivariate selection of floral trait associations. Annals of Botany 103:1557-1566.

Campbell, D. R., M. Crawford, A. K. Brody, and T. A. Forbis. 2002. Resistance to pre-dispersal seed predators in a natural hybrid zone. Oecologia (Berlin) 131:436-443.

Campbell, D. R., A. Faidiga, and G. Trujillo. 2018. Clines in traits compared over two decades in a plant hybrid zone. Annals of Botany 122:315-324.

Campbell, D. R. and K. Halama. 1993. Resource and pollen limitations to lifetime seed production in a natural plant population. Ecology 74:1043-1051.

Campbell, D. R. and J. M. Powers. 2015. Natural selection on floral morphology can be influenced by climate. Proceedings of the Royal Society B 282:21050178.

Campbell, D. R., M. V. Price, N. M. Waser, R. E. Irwin, and A. K. Brody. 2022c. Comparative impacts of long term trends in snowmelt and species interactions on plant population dynamics. Journal of Ecology 110:1102-1112.

Campbell, D. R., N. M. Waser, and E. J. Meléndez-Ackerman. 1997. Analyzing pollinator-mediated selection in a plant hybrid zone: hummingbird visitation patterns on three spatial scales. American Naturalist 149:295-315.

Campbell, D. R., N. M. Waser, M. V. Price, E. Lynch, and R. Mitchell. 1991. Components of phenotypic selection: pollen export and flower corolla width in *Ipomopsis aggregata*. Evolution 45:1458-1467.

Derryberry, E. P., Derryberry, G. E., Maley, J. M.,and R. T. Brumfield. 2014. Hzar: hybrid zone analysis using an R software package. Molecular Ecology Resources 14:652-663.

Grant, P. R. and B. R. Grant. 2002. Unpredictable evolution in a 30 year study of Darwin's finches. Science 296:707-711.

Meléndez-Ackerman, E. J. and D. R. Campbell. 1998. Adaptive significance of flower color and inter trait correlations in an *Ipomopsis* hybrid zone. Evolution 52:1293-1303.

Powers, J. M., H. M. Briggs, R. Dickson, X. Li, and D. R. Campbell. 2022. Earlier snowmelt and reduced summer precipitation alter floral traits important to pollination. Global Change Biology 28:323-339.
